# Evaluating Gulf Cooperation Council Trauma Care Infrastructure: A Scoping Review of Key Components and Gaps

**DOI:** 10.1002/wjs.70019

**Published:** 2025-07-29

**Authors:** Lubna Khan, Abbas Karim, Hachem Bey, Saud Naji Alzaid, Hani Ahmed Al‐Qadhi, Yousif Habib Alabboudi, Ruben Peralta, Husham Bakry, Thamer Nouh, Shahin Mohseni, Hassan Mashbari

**Affiliations:** ^1^ Baylor College of Medicine Houston Texas USA; ^2^ University of Texas Medical Branch Galveston Texas USA; ^3^ Department of Surgery College of Medicine Kuwait University Kuwait Kuwait; ^4^ Sultan Qaboos University Hospital Muscat Oman; ^5^ Mohamed Bin Rashid University Dubai UAE; ^6^ Hamad General Hospital and Hamad Medical Corporation Doha Qatar; ^7^ Universidad Nacional Pedro Henríquez Urena Santo Domingo Dominican Republic; ^8^ General Surgery King Hamad University Hospital Busaiteen Bahrain; ^9^ King Saud University Riyadh Saudi Arabia; ^10^ Jahra Governance Al Jahra Hospital Jahra Kuwait; ^11^ School of Medical Sciences Orebro University Orebro Sweden; ^12^ Jazan University Jazan Saudi Arabia

**Keywords:** injury, prehospital care, rehabilitation, trauma care delivery, trauma systems

## Abstract

**Background:**

Trauma systems are multifaceted frameworks that optimize patient care and outcomes. The development of trauma systems has been a regional priority in the Gulf Cooperation Council (GCC), yet implementation varies across countries. These variations contribute to measurable differences in system performance and patient outcomes. A systematic mapping of these disparities can guide efforts to harmonize standards and enhance trauma‐care delivery throughout the region.

**Methods:**

A scoping review was conducted per PRISMA‐ScR guidelines. PubMed, Scopus, and the Cochrane Library databases were searched for English‐language publications (2000–2024) on prehospital emergency care, hospital‐based trauma management, or post‐hospital rehabilitation in GCC countries. Two reviewers independently screened and charted eligible studies; articles addressing only clinical outcomes without system‐level discussion were excluded. Gray literature sources included Ministry of Health (MOH) websites, local news reports, and expert opinion.

**Results:**

Of 1758 studies, 51 were fully screened, and 43 met the inclusion criteria. All GCC countries, except for UAE, operate a single centralized EMS system via a uniform national emergency number. Fleet sizes range from 36 ambulances in Bahrain to over 1379 in Saudi Arabia, with mean response times ranging from 5.3 min in Qatar to 15 min nationally in Kuwait. Formal trauma centers are limited in the region: Bahrain has no formal trauma centers, Qatar and Kuwait each have one dedicated trauma center (level 1 and 2 equivalent, respectively), Oman has two (level 2 and level 3 equivalent), Saudi Arabia has two (level 1 equivalent), and the UAE has nine (levels 1–3 equivalent). Local trauma registries exist in all countries, with a national trauma registry only in Qatar. Posthospital rehabilitation, although variable in resources, is delivered through MOH networks in all countries and supplemented by private providers.

**Conclusion:**

Despite progress, gaps persist in trauma center accreditations, national registry development, and formation of integrated rehabilitation networks. Concerted improvements could further enhance trauma care delivery in the region with a desired improvement in overall outcomes.

## Graphical abstract

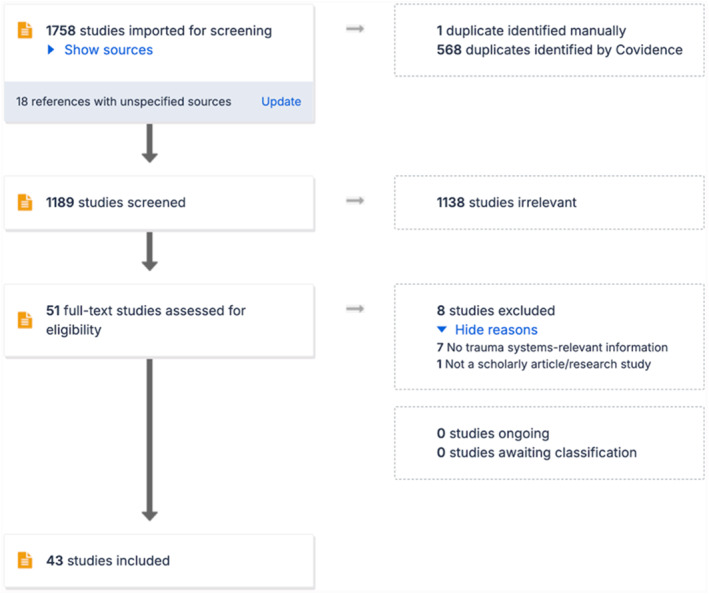

**Abbreviations**
CBAHI: Central Board for Accreditation of Healthcare Institutions
EMS: Emergency Medical Services
EMTs: Emergency Medical Technicians
GCC: Gulf Cooperation Council
MOH: Ministry of Health
UAE: United Arab Emirates

## 1 Introduction

The Gulf Cooperation Council (GCC), which includes Bahrain, Kuwait, Oman, Qatar, Saudi Arabia, and the United Arab Emirates (UAE), is a rapidly growing region. Its total population is projected to increase from 56.4 million in 2018 to nearly 66 million by 2030, a 17% rise over 12 years [1]. Urban populations alone are expected to grow by approximately 30% between 2020 and 2030 [2]. Rapid urbanization has contributed to higher injury rates, with trauma being a leading cause of death [3, 4]. In response, GCC countries have emphasized the need to adopt best practices in trauma system development, align with international standards, and invest strategically in health infrastructure. For instance, Saudi Arabia's Vision 2030 calls for healthcare modernization through upgrades to trauma care systems, investments in emergency services, and the creation of integrated trauma networks aimed at improving outcomes [5, 6]. Qatar and Oman have launched Vision 2040 grounded in the principle of “Health for All” with a focus on updating their national health policies and strengthening areas of collaboration with various sectors [7, 8]. United Arab Emirates has prioritized enhancements in emergency medical services and trauma care, committing to improve prehospital care and develop trauma centers that meet global benchmarks [9]. Health ministries in Kuwait and Bahrain have similarly acknowledged the need for organized trauma care by modernizing emergency medical services (EMS), establishing trauma registries, and implementing coordinated management policies [10, 11, 12].

Despite these national initiatives and investments, significant gaps persist in trauma system design, organization, and intercountry learning across the GCC. Although individual country studies describe elements of prehospital care, trauma centers, registries, and rehabilitation, no prior synthesis has mapped these components across the entire region. To address this, our scoping review systematically explores existing models of care, accreditation frameworks, registry implementation, and rehabilitation networks. Given the exploratory nature of our questions, a scoping approach was deemed ideal. Our review focuses on three main domains [1]: prehospital emergency care, including the organization, fleet composition, dispatch models, paramedic training, and response times [2]; hospital‐based trauma management, with attention to trauma‐center designations, accreditation frameworks, trauma registry implementation (distinguishing between facility‐level pilots and national databases); and [3] posthospital rehabilitation, exploring the structure, accessibility, and integration of rehabilitation services into the broader trauma care continuum. By charting heterogeneous evidence to clarify system‐level data and identify gaps, this review lays the groundwork for targeted research, policy harmonization, and collaborative system strengthening.

## 2 Methods

A scoping review was conducted to map trauma care delivery across the six GCC countries, guided by the Arksey and O'Malley framework and Joanna Briggs Institute methodology and reported in accordance with the PRISMA extension for scoping reviews (PRISMA‐ScR) [13]. PubMed (Medline), Scopus, and the Cochrane Library (Trials) databases were searched for peer‐reviewed studies written in the English language published between January 2000 and February 2024. This timeframe captures both the early development of formal trauma systems in the GCC and the most recent evidence on infrastructure and policy. English‐language publications were prioritized as medical education and scientific dissemination in the GCC are predominantly conducted in English. Included sources focused on prehospital emergency medical services, hospital‐based trauma management, or posthospital care in Bahrain, Kuwait, Oman, Qatar, Saudi Arabia, and the UAE. Exclusion criteria comprised sources focused solely on individual clinical outcomes without system‐level discussion, conference abstracts lacking full‐text manuscripts, and publications in languages other than English. In addition, each country was represented by one active trauma surgeon who is directly involved in trauma system improvement on a national level, serving as a co‐investigator in this study. Ministries of health were not directly consulted during the synthesis or interpretation of findings.

We applied a three‐stage search strategy. First, an initial limited search was performed in the PubMed and Scopus databases to identify key index terms and subject headings. Next, a full search was executed across all three databases on February 28, 2024, using controlled vocabulary and free‐text terms for trauma system, emergency medical services, trauma center, operating theater and blood bank, and subspecialty services, combined with country names. Full search syntax is provided in Supporting Information S1: Appendix A. Gray literature sources included Ministry of Health (MOH) websites, local news reports, and regional expert opinion.

All citations were uploaded to Covidence for screening (www.covidence.org). Two reviewers (AK and HB) independently assessed titles and abstracts and conducted full‐text review against predefined eligibility criteria. Discrepancies were resolved by consensus. For data charting, we used a standardized form capturing publication details (author, year, and country), system component(s) addressed, key findings, and gaps. Extracted data were collated by domain: prehospital services, hospital management, trauma registry implementation, and availability of post‐hospital care, and summarized in tables and narrative text. We used backward citation tracking and author tracking to identify additional articles not captured in the initial database search.

Although the review was completed prior to registration, the full protocol was retrospectively registered on the Open Science Framework (DOI 10.17605/OSF.IO/HVPJQ) on May 1, 2025, to enhance transparency and provide a public record of our a priori methods. As this review used only publicly available data, ethics approval was not required.

## 3 Results

**FIGURE 1:**
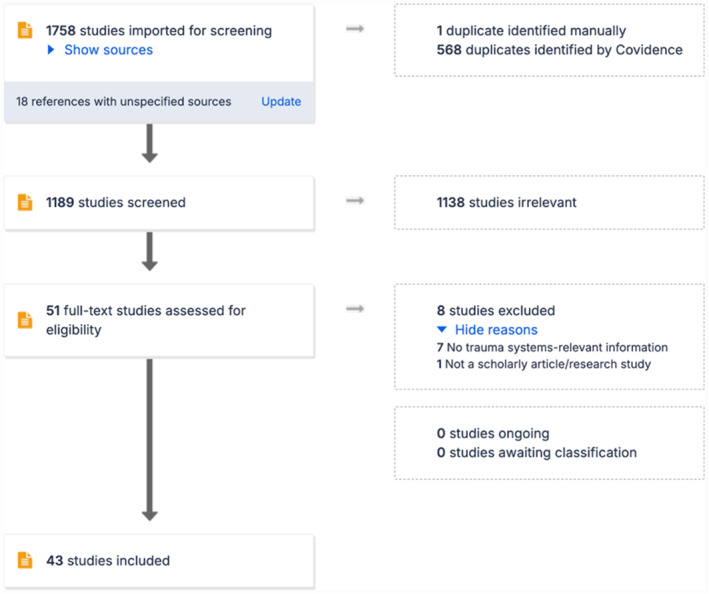
PRISMA‐ScR flow diagram illustrating study selection for the scoping review.

A PRISMA‐ScR flow diagram (Figure 1) documents the source selection process. After screening titles and abstracts, 51 full‐text articles were assessed and eight were excluded for lack of relevant system information or non‐scholarly format. The final review included 43 studies.

Saudi Arabia contributed the largest share (17 studies), followed by Oman [8], the UAE [6], Qatar [5], Bahrain [2], and Kuwait [2], with three additional mixed‐country analyses. Data regarding pre‐hospital infrastructure (*n* = 31), hospital‐based trauma care (*n* = 35), and post‐hospital rehabilitation (*n* = 9) was extracted from these articles. Breakdown of included articles by country and phase of care (prehospital, hospital, and posthospital) is presented in Table 1. Relevant articles identified outside of the scoping review are listed in Supporting Information S1: Appendix B. Table 2A, 2B provide summary of the prehospital infrastructure, hospital trauma care, and posthospital rehabilitation services across the GCC countries.

**TABLE 1:** Mapping of trauma care delivery components across reviewed articles.

#	Reference	Country	Article title	Pre‐hospital infrastructure	Hospital trauma care	Post‐hospital rehabilitation
1	Liu 2021	**Bahrain**	Epidemiology of pediatric trauma in the Kingdom of Bahrain: a National pediatric trauma registry pilot study	X	X	
2	Abuzeyad 2018	**Bahrain**	Emergency medicine in the Kingdom of Bahrain	X	X	
3	Sabah 2018	**Kuwait**	The aftermath of the Kuwait mosque bombing: A retrospective cohort analysis and lessons learned	X	X	
4	Heshmat 2015	**Kuwait**	Development of tertiary cardiac care center in Kuwait: Sabah Al‐Ahmad cardiac center model		X	
5	Al Balushi 2022	**Oman**	Evaluating trauma care capabilities using the essential trauma care guidelines of the world health organization: Cross‐sectional study of primary health centers in Muscat, Oman	X	X	
6	Mehmood 2018	**Oman**	Childhood injuries in Oman: Retrospective review of a multicenter trauma registry data	X	X	
7	Mehmood 2017	**Oman**	Trauma care in Oman: A call for action	X	X	X
8	Al‐Kindi 2017	**Oman**	Distribution of trauma care facilities in Oman in relation to high‐incidence road traffic injury sites: Pilot study	X	X	
9	Al‐Kashmiri 2017	**Oman**	Trauma care in Oman: Where do we stand and where should we be heading?	X	X	
10	Joshi 2010	**Oman**	Development of blood transfusion service in sultanate of Oman		X	
11	Al‐Azri 2009	**Oman**	Emergency medicine in Oman: Current status and future challenges	X	X	
12	Al‐Shaqsi 2009	**Oman**	EMS in the sultanate of Oman	X		
13	Al‐Thani 2021	**Qatar**	Emergency medical services (EMS) transportation of trauma patients by geographic locations and in‐hospital outcomes: Experience from Qatar	X	X	
14	El‐Menyar 2020	**Qatar**	Maturation process and international accreditation of trauma system in a rapidly developing country	X	X	X
15	Al‐Thani 2019	**Qatar**	Evolution of the Qatar trauma system: The journey from inception to verification	X	X	X
16	Williams 2015	**Qatar**	Improving prehospital care around the world. How three countries are improving quality and innovating paramedic service	X		
17	Al‐Dulimi 2009	**Qatar**	Trauma mortality in the state of Qatar, 2006–2007	X	X	
18	Al‐Otaibi 2023	**Saudi Arabia**	The characteristics and distribution of emergency medical services in Saudi Arabia	X		
19	Chowdhury 2022	**Saudi Arabia**	Development of the Saudi Arabian trauma system		X	
20	Alsenani 2021	**Saudi Arabia**	Comparison of trauma management between two major trauma services in Riyadh, Kingdom of Saudi Arabia and Melbourne, Australia		X	X
21	Aljabri 2021	**Saudi Arabia**	Hospital pharmacy preparedness and pharmacist role during disaster in Saudi Arabia		X	
22	Alshamrani 2020	**Saudi Arabia**	Current state of trauma services in Saudi Arabia	X	X	X
23	Alharbi 2020	**Saudi Arabia**	Current trauma care system in Saudi Arabia: A scoping literature review	X	X	X
24	Ford 2020	**Saudi Arabia**	Experience gained from the implementation of the Saudi TraumA registry (STAR)		X	
25	Khattab 2019	**Saudi Arabia**	Emergency medicine in Saudi Arabia: a Century of progress and a bright vision for the future	X	X	X
26	Alotaibi 2019	**Saudi Arabia**	Assessing the pre‐hospital care preparedness to face mass casualty incident in Saudi Arabia in 2017–2018	X		
27	AlShammari 2017	**Saudi Arabia**	Evolution of emergency medical services in Saudi Arabia	X	X	
28	Al‐Shareef 2017	**Saudi Arabia**	Evaluation of hospitals’ disaster preparedness plans in the holy city of Makkah (Mecca): A cross‐sectional observation study		X	
29	Alghnam 2014	**Saudi Arabia**	Burden of traumatic injuries in Saudi Arabia: Lessons from a major trauma registry in Riyadh, Saudi Arabia		X	
30	Elkum 2011	**Saudi Arabia**	Canadian emergency department triage and acuity scale: Implementation in a tertiary care center in Saudi Arabia	X	X	
31	Al‐Naami 2010	**Saudi Arabia**	Trauma care systems in Saudi Arabia: An agenda for action	X	X	X
32	Qureshi 2010	**Saudi Arabia**	Triage systems: A Review of the literature with reference to Saudi Arabia	X		
33	Al‐Yousuf 2002	**Saudi Arabia**	Organization of the Saudi health system			X
34	Al Naami 2001	**Saudi Arabia**	Evaluation of trauma registry data in Asir region	X	X	
35	Alao 2020	**UAE**	Trauma system developments reduce mortality in hospitalized trauma patients in Al‐Ain city, United Arab Emirates, despite increased severity of injury	X	X	
36	Fares 2014	**UAE**	Emergency medicine in the United Arab Emirates	X	X	
37	Shaban 2010	**UAE**	Toward a national trauma registry for the United Arab Emirates		X	
38	Partridge 2009	**UAE**	Emergency medicine in Dubai, UAE	X	X	
39	Shaban 2009	**UAE**	The long term effects of early analysis of a trauma registry		X	
40	Sasser 2004	**UAE**	Prehospital emergency care in Abu Dhabi, United Arab Emirates	X		
41	Alsubahi 2024	**Mixed**	Healthcare quality from the perspective of patients in gulf cooperation council countries: A systematic literature review	X	X	
42	Moafa 2019	**Mixed**	What is known about the quality of out‐of‐hospital emergency medical services in the Arabian gulf states? A systematic review	X		
43	Choi 2017	**Mixed**	Comparison of trauma care systems in Asian countries: A systematic literature review	X	X	

**TABLE 2A:** Prehospital infrastructure in GCC countries.

Country	Prehospital			
	EMS provider	Call number	Ambulance/Air transport	EMS response times
Bahrain	National ambulance under ministry of interior.	999	13 satellite ambulance stations with 36 ambulances and 2 helicopters.	11.5 min.
Kuwait	Emergency medical services directorate under ministry of health.	112	86 ambulance dispatch stations with 200 ground ambulances, +air medical transport.	10–15 min.
Qatar	Hamad medical corporation ambulance service (HMCAS) under Hamad medical corporation (HMC), a non‐profit organization.	999	200 ambulances, 22 rapid response vehicles, 16 bicycles, and 3 helicopters.	5.3 min (urban), 6.4 min (rural).
Saudi Arabia	Saudi Red Crescent under government‐funded Saudi Red Crescent Authority.	997	1379 ambulances, 14 helicopters across 486 dispatch centers.	10–13 min.
Oman	National ambulance “Isa’af” under Royal Oman Police & ministry of health.	9999	23 permanent ambulance stations with an additional five seasonal units, +air medical transport.	< 10 min (urban), 20 min (rural).
United Arab Emirates	The centre of ambulance services (Dubai) under police, national ambulance (Northern Emirates) under national guard command.	999 (Dubai); 998 (Northern Emirates)	> 110 ambulances across > 50 ambulance stations, 1 helicopter.	Not available.

**TABLE 2B:** Hospital trauma care and posthospital rehabilitation in GCC countries.

Country	Hospital		Posthospital care
	Trauma centers	Trauma registry	
Bahrain	Salmaniya medical complex (SMC), Bahrain defence force hospital (BDFH), King Hamad university hospital (KHUH).	Facility‐level pediatric trauma registry (short‐term).	Primarily through the ministry of health’s network of primary health centers.
Kuwait	Sheikh Jaber Al Ahmad Al Jaber Al Sabah hospital, Al‐Adan hospital, Al‐Jahra hospital, Mubarak Al‐Kabeer hospital, Sabah hospital.	Combined facility‐level trauma registry at 3 trauma centers.	Ministry of health–funded facilities operate across six regions and are supplemented by private hospitals and clinics.
Qatar	Hamad general hospital (HGH): Tertiary referral hospital, Hamad trauma center (HTC).	National trauma registry (Hamad medical corporation trauma registry).	National Qatar Rehabilitation Institute for rehabilitation.
Saudi Arabia	King Saud medical city (KSMC), King Abdulaziz medical city (KAMC).	Facility‐level registries (KAMC trauma registry and Saudi trauma registry at KSMC).	Rehabilitation services available at Ministry of Health hospitals across all 13 administrative regions.
Oman	Sultan Qaboos university hospital, Khoula hospital.	Facility‐level registries (at Khoula and Nizwa hospitals).	Tertiary centers offer rehabilitation.
United Arab Emirates	Tawam hospital (Abu Dhabi), Al Ain hospital (Abu Dhabi), Sheikh Shakhbout medical city (SSMC) (Abu Dhabi), Sheikh Khalifa medical city (SKMC) (Abu Dhabi), Rashid hospital trauma centre (Dubai), Jebel Ali emergency and trauma centre (Dubai).	Facility‐level trauma registry (Abu Dhabi trauma registry).	Mix of several government and private providers.

### 3.1 Bahrain

#### 3.1.1 Prehospital Care

Bahrain's national prehospital service, National Ambulance, was launched in 2019 and operates under the Ministry of Interior. It provides free coverage via the nationwide emergency number 999 [14]. A centralized call center coordinates dispatch of 36 ambulances across 13 dispatch stations while tertiary hospitals manage ambulance deployment [14, 15]. EMTs are trained in both basic and advanced life support, and all patients receive ALS services [15]. Their system follows the Anglo‐American “load‐and‐go” approach, with average reported response time of 11.5 min [15, 16]. Two helicopters are available for air medical transport [16].

#### 3.1.2 Hospital Care

All trauma care is provided at the three major tertiary care centers: Salmaniya Medical Complex (main trauma center), Royal Medical Services of Bahrain Defense Force (designated burn center), and King Hamad University Hospital [15]. There is no trauma center designation framework or national registry, but a facility‐level database was used to analyze 3 months of pediatric trauma data [17].

#### 3.1.3 Posthospital Care

Rehabilitation services are provided through the MOH's network of primary health centers [18]. Major public hospitals also maintain inpatient and outpatient rehabilitation units.

### 3.2 Kuwait

#### 3.2.1 Prehospital Care

Kuwait's national prehospital service, EMS Directorate, was launched in 1988 and operates under the MOH [14]. It provides free emergency coverage via the nationwide emergency number 112 [14]. A centralized control center coordinates dispatch of 200 ambulances across 86 dispatch stations using an electronic triage system, achieving an average response time of 10–15 min [19, 20]. EMTs are licensed by the MOH and trained in both basic and advanced life support [21]. Air medical transport is also available [14, 19].

#### 3.2.2 Hospital Care

Trauma care is provided at several hospitals: Sheikh Jaber Al Ahmad Al Jaber Al Sabah hospital, Al‐Adan hospital, Al‐Jahra hospital, Mubarak Al‐Kabeer hospital, and Sabah hospital [22, 23]. Al‐Adan Hospital has been classified as a Level 2 center in the literature, and as of 2014, Kuwait had no Level 1 equivalent trauma center [24, 25]. There is no trauma center designation framework or national registry; however, a combined local trauma registry is maintained at Sheikh Jaber Al Ahmad Al Jaber Al Sabah Hospital, Al Adan Hospital, and Al Jahra Hospital.

#### 3.2.3 Posthospital Care

Physical therapy in Kuwait is government‐funded through the MOH, with additional services offered by private hospitals and clinics [25].

### 3.3 Qatar

#### 3.3.1 Prehospital Care

Qatar's national prehospital service, Hamad Medical Corporation Ambulance Service, was launched in 2008 and operates under Hamad Medical Corporation, a nonprofit overseen by the Ministry of Public Health [26]. It provides free emergency coverage via the nationwide emergency number 999 [26, 27]. Dispatch follows a hub‐and‐spoke model with six regional hubs, each supported by 5–7 spoke stations [28]. The fleet includes 200 ambulances, 22 rapid response vehicles, 16 bicycles, and 3 helicopters [29]. All emergency calls are answered by a two‐crew paramedic ambulance trained in both basic and advanced life support, with critical care trained EMTs mobilized for complex cases [26]. Median reported EMS response times are 5.3 minutes in urban areas, 6.4 minutes in rural areas, and 55 minutes for remote cases requiring air medical transport [29, 30].

#### 3.3.2 Hospital Care

All major trauma cases are transferred to Hamad Trauma Center, the trauma unit within the main tertiary referral hospital, Hamad General Hospital, whereas minor injuries are managed at the nearest facility within the patient's municipality [26, 29]. Although Qatar does not have a national trauma‐center accreditation program, voluntary international recognitions (American College of Surgeons Committee on Trauma consultative review noting compliance with Level I standards and Trauma Distinction Award from Accreditation Canada International) serve as de facto benchmarks for trauma‐center designation [26, 29, 31]. The Hamad Medical Corporation Trauma Registry, established in 2007, functions as the national trauma registry and is managed by dedicated trauma registrars and data analysts [26, 32].

#### 3.3.3 Posthospital Care

The Qatar Rehabilitation Institute, part of Hamad Medical Corporation, provides comprehensive inpatient and outpatient rehabilitation services [30].

### 3.4 Saudi Arabia

#### 3.4.1 Prehospital Care

Saudi Arabia's national prehospital service, Saudi Red Crescent Authority, was founded in 1963 and operates under the authority of the Council of Ministers as an independent government agency [6]. It provides free emergency coverage via the nationwide emergency number 997 [33, 34]. A central command center coordinates the dispatch of 1379 ambulances across 486 centers and uses an electronic triage system to forward calls to regional dispatch units, which assign the nearest available crew [35]. All emergency calls are answered paramedics trained in basic life support, with mobile intensive care units staffed by paramedics and physicians mobilized for complex cases to provide advanced life support [36]. Patients are often transported to the nearest hospital regardless of trauma care capabilities [6, 37]. Reported response times average 10–13 min [20]. Fourteen helicopters are available for air medical transport [35].

#### 3.4.2 Hospital Care

There are only two documented trauma centers in Saudi Arabia that closely align with ACS Level 1 standards: King Saud Medical City and King Abdulaziz Medical City, both located in Riyadh [5, 6, 35, 38, 39]. Major trauma in the rest of the country is managed by tertiary hospitals [35]. To guide the rollout of new trauma networks, the Saudi Central Board for Accreditation of Healthcare Institutions (CBAHI) developed certification requirements for trauma centers classifying facilities into Levels 1–3 [4].

There are two facility‐level trauma registries in Saudi Arabia: one at King Abdulaziz Medical City, established in 2001 and modeled after the National Trauma Data Bank, and the Saudi Trauma Registry, piloted at King Saud Medical City in 2017 using the Global Alliance for Care of the Injured Minimum Dataset [39, 40].

#### 3.4.3 Posthospital Care

Rehabilitation is provided at MOH hospitals, with most offering physical therapy and larger centers also providing occupational therapy, speech therapy, and prosthetic services [41].

### 3.5 Oman

#### 3.5.1 Prehospital Care

Oman's national ambulance service, *Isa'af*, was launched in 2004 and operates under the Royal Oman Police in coordination with the MOH [42, 43]. It provides coverage for a fee via the nationwide emergency number 9999 [42, 43]. A central dispatch center uses an electronic system to coordinate dispatch from 23 stations and five seasonal units to the nearest facility [42]. All emergency calls are answered by a two‐crew paramedic ambulance trained in both basic and advanced life support [42, 44]. Reported response times average under 10 min in urban areas and 20 min in rural regions [42]. Oman Air Ambulance facilitates patient transfers from other major cities to medical facilities in Muscat [45].

#### 3.5.2 Hospital Care

Sultan Qaboos University Hospital and Khoula Hospital in Muscat are considered the highest‐level trauma centers in Oman, with capacities equivalent to ACS Level 2 and Level 3 trauma centers, respectively [44, 46]. Primary care centers serve as first‐contact points for rural emergencies, but emergency preparedness varies because of gaps in equipment and training [42, 48]. Although heath care administration is centralized and delivered through the MOH, there is no formal trauma accreditation framework [46, 49].

There is no national trauma registry [49]; however, a pilot project at Khoula and Nizwa hospitals demonstrated the feasibility of implementing a mobile‐based electronic trauma registry at the national level [44, 48].

#### 3.5.3 Posthospital Care

Rehabilitation services are primarily delivered through MOH tertiary centers, with additional support from community‐based and private rehabilitation facilities [50].

### 3.6 United Arab Emirates

#### 3.6.1 Prehospital Care

In the UAE, EMS dispatch systems differ between Abu Dhabi/Northern Emirates and Dubai. In Abu Dhabi and the Northern Emirates, EMS is provided by National Ambulance, launched in 2010 under the National Guard Command [51]. It provides free coverage via the emergency number 998 [51]. A police‐run control center uses an electronic dispatch system to triage calls and coordinate dispatch across 60 ambulance sites in five emirates [51]. EMTs are trained in both basic and advanced life support [51]. Crews coordinate with civil defense and the Ministry of Interior Air Wing for complex or remote responses [51, 52].

In Dubai, EMS is provided by the Dubai Corporation for Ambulance Services (DCAS) under the regulatory oversight of the Dubai Health Authority [53]. It provides free coverage via the emergency number 999 [53]. The DCAS control center uses an electronic dispatch system to triage calls and coordinate dispatch of 177 ambulances across 68 stations [54]. All emergency calls are answered by a two‐crew paramedic ambulance trained in both basic and advanced life support [54]. Air medical transport via police helicopters is also available [55].

#### 3.6.2 Hospital Care

Nine hospitals in the UAE provide trauma care, including seven in Abu Dhabi and two in Dubai. In Abu Dhabi, Tawam Hospital, Al Ain Hospital, Sheikh Shakhbout Medical City, and Sheikh Khalifa Medical City are tertiary centers, whereas Al Rahba Hospital, Al Gharbia hospital, and Al Ruwais hospitals provide secondary care [56]. In Dubai, trauma care is provided at Rashid Hospital Trauma Center and Jebel Ali Emergency and Trauma Center [55, 57].

Abu Dhabi and Dubai have their own health authorities responsible for licensing and regulating trauma center accreditation using formal designation standards [58]. In Abu Dhabi, the Department of Health aims to implement its “Standard for Trauma Center Level I” and companion Level II/III criteria to accredit hospitals based on service specifications, staffing, and performance requirements [59]. Dubai Health Authority has similarly published a formal trauma‐center designation guideline [60]. However, no hospital has yet met these standardized benchmarks.

There is no national trauma registry in the UAE, but facility‐level registries are maintained in Abu Dhabi and Dubai. The Abu Dhabi Trauma Registry, established in 2014, is based on the National Trauma Data Bank dataset [61, 62, 63]. Rashid Hospital in Dubai has participated in Germany's TraumaNetzwerk DGU registry since 2011 [64].

#### 3.6.3 Posthospital Care

Rehabilitation services in the UAE are delivered through a mix of government and private providers [65].

## 4 Discussion

This scoping review presents the first comprehensive mapping of trauma care infrastructure across the GCC region. It highlights strengths in prehospital coverage and accessibility while also identifying variability in trauma center accreditation, data system development, and integration of rehabilitation services. These findings reveal both progress and opportunities for system improvement across the region.

Prehospital care in the GCC is centrally managed in most countries, except the UAE, with minimal‐to‐no fees for service, and ability to provide basic and advanced life support [14]. Response times are generally acceptable in urban areas, though lengthen considerably in rural and remote areas, highlighting the need for expanded coverage and more frequent use of helicopter EMS [66]. Fleet sizes and deployment models vary significantly across countries, and more studies are needed to determine which configurations are most financially sustainable and effective in providing timely access to care [67]. Although most countries have standardized electronic dispatch protocols, few studies have assessed adherence to these protocols [68].

Hospital‐based trauma care is limited and unevenly distributed across the GCC region. The UAE has nine trauma centers concentrated in just two of its seven Emirates. Saudi Arabia has two centers, and both are located in Riyadh. Oman has two centers nationwide, whereas Kuwait and Qatar each have one. Bahrain has no formally documented trauma center. This limited distribution leaves large areas without direct access to specialist trauma teams, increasing the risk of preventable mortality [69]. Adopting a tiered trauma center designation framework, such as the three‐level models developed by CBAHI or Abu Dhabi, can support other countries in scaling capacity and aligning resources with community needs [70].

Trauma data collection is essential for quality improvement, yet only one GCC country maintains a national trauma registry [31, 71]. Whereas facility‐level registries were piloted in several hospitals across the GCC, national data systems are yet to be implemented. Key barriers include the absence of a mandatory reporting framework, fragmented data collection across the phases of care, and reliance on short‐term funding that disrupts continuity when grants end [35, 61, 72]. Ensuring external validity is critical for maintaining data quality, yet limited trained personnel, low clinician engagement, and data privacy concerns further complicate efforts to scale registries beyond individual facilities [61, 72].

Posthospital rehabilitation is universally acknowledged as essential for optimizing long‐term outcomes, yet service integration varies across countries in terms of availability, scope, and coordination within national health systems [6, 49]. In most GCC countries, rehabilitation is delivered through MOH networks augmented by specialized institutes and private centers. However, smaller hospitals often lack onsite occupational or speech therapy and rely on external referrals [25].

Moving forward, a coordinated regional strategy is highly recommended. First, narrowing rural–urban response gaps will require strengthening prehospital networks through expanded station coverage, air medical transport, and telemedicine support. Second, establishing clear criteria for trauma center designation, paired with a regulatory body to conduct regular audits, will support expansion beyond capital cities, thereby reducing the morbidity and mortality burden. Third, creating a unified Gulf Trauma Registry, building on existing pilots and leveraging digital tools, will enable performance monitoring and guide resource allocation [73]. Finally, integrating rehabilitation into the trauma care continuum through regional guidelines, workforce development, and investment in community programs will help optimize long‐term recovery [74].

Actionable priorities can be tailored to different stakeholder levels such as: ministries should formalize trauma center accreditation with standardized criteria and develop unified national trauma registries for auditing and quality improvements. Hospitals can prioritize staff certification, implement facility‐level registries, and improve interfacility transfer protocols. Academic institutions can support by creating trauma protocols, evaluating trauma outcomes, supporting registry design, and training clinicians in trauma systems management. Coordinated efforts across these levels may accelerate system maturation and regional harmonization. These recommendations align with national development plans (such as Saudi Arabia's Vision 2030, Oman's Vision 2040, and others) which emphasize integrated patient‐centered health systems, enhanced emergency preparedness, and expanded digital health. Coordinated efforts across these levels may accelerate system maturation and regional harmonization.

This review has several limitations, including reliance on publicly available sources, inconsistent reporting across countries, and possible omission of unpublished data. The availability and depth of information varied significantly between countries, with some nations (e.g., Saudi Arabia and Qatar) having more comprehensive documentation of trauma system components than others (e.g., Bahrain and Kuwait). This unevenness limited the ability to conduct balanced inter‐country comparisons and may have led to an overrepresentation of countries with more transparent or better‐documented systems. Furthermore, differences in governance structures, terminology, and reporting formats complicated synthesis and introduced interpretive challenges. Data on private sector contributions to EMS and trauma care were particularly scarce and inconsistently reported. To address limitations in the original search strategy, citation and author tracking were used to identify additional relevant articles. Although this helped overcome search term limitations, it reduced the overall reproducibility of the search process. Despite these constraints, the synthesis highlights clear priorities for policymakers, providers, and researchers across the GCC.

Future work should involve structured consultation with ministries of health and national trauma system leads to validate our findings, contextualize gaps, and ensure policy alignment. Concurrently, research should evaluate the comparative effectiveness of prehospital dispatch models, identify local barriers to national trauma registry implementation, and assess the feasibility and cost‐effectiveness of tiered trauma center designation to guide tailored system development.

## 5 Conclusion

Despite progress in strengthening trauma systems across the GCC, key gaps remain in foundational areas. Trauma center accreditation lacks standardization, limiting quality assurance, and comparability across facilities. National trauma registries are underdeveloped, with data collection largely confined to individual institutions and lacking regional integration. Rehabilitation services are fragmented and often poorly linked to the broader continuum of care. Coordinated efforts in policy, funding, and workforce development are essential to advancing the effectiveness and equity of trauma care across the region.

## Author Contributions

**Lubna Khan:** conceptualization (lead), methodology (lead), writing – original draft (lead), formal analysis (lead), writing – review and editing (equal). **Abbas Karim, Hachem Bey:** methodology (supporting), writing – review and editing (equal). **Shahin Mohseni, Hassan Mashbari:** conceptualization (supporting), writing – original draft (supporting), writing – review and editing (equal). **Saud Naji Alzaid, Hani Ahmed Al‐Qadhi, Yousif Habib Alabboudi, Ruben Peralta, Husham Bakry, Thamer Nouh:** writing – review and editing (equal).

## Conflicts of Interest

The authors declare no conflicts of interest.

## Supporting information

Supporting Information S1

## Data Availability

The data that support the findings of this study are available from the corresponding author upon reasonable request.
