# Supplementary material for: Evaluating Gulf Cooperation Council Trauma Care Infrastructure: A Scoping Review of Key Components and Gaps
Source: World J Surg. 2025 Jul 29;49(10):2921–32. doi: 10.1002/wjs.70019 (PMC12515026; doi:10.1002/wjs.70019)
Supplement: Supplementary file 1 — Supporting Information S1 [file WJS-49-2921-s001.docx]

**Appendix A:**

**PubMed(Medline): Search ran on 2/28/24 and produced 763 results.**

("Emergency Medical Services"[Mesh] OR ("emergency care"[tw] OR "emergency health service*"[tw] OR "emergency medical service*"[tw] OR emergicenter*[tw] OR "medical emergency service*"[tw] OR "prehospital emergency care*"[tw] OR "prehospital care*"[tw] OR "pre-hospital emergency care*"[tw] OR "pre-hospital care*"[tw] OR EMS[tw]) OR "Trauma Centers"[Mesh] OR Traumatology[Mesh] OR ("trauma management*"[tw] OR "trauma system*"[tw] OR "trauma network*"[tw] OR "trauma care*"[tw] OR "trauma registr*"[tw] OR "trauma bay*"[tw]) OR "Operating Rooms"[Mesh] OR ("operating room*"[tw] OR "surgery facilit*"[tw] OR "surgical facilit*"[tw] OR "acute care surger*"[tw] OR "surgical critical care*"[tw] OR "emergency general surger*"[tw] OR "trauma surger*"[tw] OR "surgical trauma care*"[tw]) OR "Blood Banks"[Mesh] OR "blood bank*"[tw]) AND (Bahrain[Mesh] OR Kuwait[Mesh] OR Oman[Mesh] OR Qatar[Mesh] OR "Saudi Arabia"[Mesh] OR "United Arab Emirates"[Mesh] OR (bahrain[tw] OR kuwait[tw] OR oman[tw] OR muscat[tw] OR qatar[tw] OR katar[tw] OR quatar[tw] OR "saudi arabia"[tw] OR "united arab emirates"[tw] OR "trucial states"[tw] OR "abu dhabi"[tw] OR UAE[tw] OR "arabian gulf states"[tw] OR "middle eastern gulf nations"[tw])) Filters: from 2000 - 2024

**Cochrane Trials: Search ran on 2/28/24 and produced 18 results.**

1. [mh "Emergency Medical Services"] OR ("emergency care":ti,ab,kw OR ("emergency health" NEXT service*):ti,ab,kw OR ("emergency medical" NEXT service*):ti,ab,kw OR emergicenter*:ti,ab,kw OR ("medical emergency" NEXT service*):ti,ab,kw OR ("prehospital emergency" NEXT care*):ti,ab,kw OR ("prehospital" NEXT care*):ti,ab,kw OR ("pre-hospital emergency" NEXT care*):ti,ab,kw OR ("pre-hospital" NEXT care*):ti,ab,kw OR EMS:ti,ab,kw)

2. [mh "Trauma Centers"] OR [mh Traumatology] OR (("trauma" NEXT management*):ti,ab,kw OR ("trauma" NEXT system*):ti,ab,kw OR ("trauma" NEXT network*):ti,ab,kw OR ("trauma" NEXT care*):ti,ab,kw OR ("trauma" NEXT registr*):ti,ab,kw OR ("trauma" NEXT bay*):ti,ab,kw)

3. [mh "Operating Rooms"] OR (("operating" NEXT room*):ti,ab,kw OR ("surgery" NEXT facilit*):ti,ab,kw OR ("surgical" NEXT facilit*):ti,ab,kw OR ("acute care" NEXT surger*):ti,ab,kw OR ("surgical critical" NEXT care*):ti,ab,kw OR ("emergency general" NEXT surger*):ti,ab,kw OR ("trauma" NEXT surger*):ti,ab,kw OR ("surgical trauma" NEXT care*):ti,ab,kw)

4. [mh "Blood Banks"] OR ("blood" NEXT bank*):ti,ab,kw

5. #1 OR #2 OR #3 OR #4

6. [mh Bahrain] OR [mh Kuwait] OR [mh Oman] OR [mh Qatar] OR [mh "Saudi Arabia"] OR [mh "United Arab Emirates"] OR (bahrain:ti,ab,kw OR kuwait:ti,ab,kw OR oman:ti,ab,kw OR muscat:ti,ab,kw OR qatar:ti,ab,kw OR katar:ti,ab,kw OR quatar:ti,ab,kw OR "saudi arabia":ti,ab,kw OR "united arab emirates":ti,ab,kw OR "trucial states":ti,ab,kw OR "abu dhabi":ti,ab,kw OR UAE:ti,ab,kw OR "arabian gulf states":ti,ab,kw OR "middle eastern gulf nations":ti,ab,kw)

7. #5 AND #6

8. Custom Year Range: 2000-2024

**Scopus: Search ran on 2/28/24 and produced 977 results.**

INDEXTERMS ( "Emergency Medical Services" ) OR TITLE-ABS-KEY ( "emergency care" OR "emergency health service*" OR "emergency medical service*" OR emergicenter* OR "medical emergency service*" OR "prehospital emergency care*" OR "prehospital care*" OR "pre-hospital emergency care*" OR "pre-

hospital care*" OR ems ) OR INDEXTERMS ( "Trauma Centers" ) OR INDEXTERMS ( traumatology ) OR TITLE-ABS-KEY ( "trauma management*" OR "trauma system*" OR "trauma network*" OR "trauma care*" OR "trauma registr*" OR "trauma bay*" ) OR INDEXTERMS ( "Operating Rooms" ) OR TITLE-ABS-KEY ( "operating room*" OR "surgery facilit*" OR "surgical facilit*" OR "acute care surger*" OR "surgical critical care*" OR "emergency general surger*" OR "trauma surger*" OR "surgical trauma care*" ) OR INDEXTERMS ( "Blood Banks" ) OR TITLE-ABS-KEY ( "blood bank*" ) AND INDEXTERMS ( bahrain ) OR INDEXTERMS ( kuwait ) OR INDEXTERMS ( oman ) OR INDEXTERMS ( qatar ) OR INDEXTERMS ( "Saudi Arabia" ) OR INDEXTERMS ( "United Arab Emirates" ) OR TITLE-ABS-KEY ( bahrain OR kuwait OR oman OR muscat OR qatar OR katar OR quatar OR "saudi arabia" OR "united arab emirates" OR "trucial states" OR "abu dhabi" OR uae OR "arabian gulf states" OR "middle eastern gulf nations" ) AND PUBYEAR > 1999 AND PUBYEAR < 2025

| **Appendix B. Component Mapping of Trauma Care Delivery in Supplementary Articles** | | | | | | | |
| --- | --- | --- | --- | --- | --- | --- | --- |
| **#** | **Reference** | **Country** | **Article Title** | **Pre-hospital Infrastructure** | **Hospital Trauma Care** | **Post-hospital Rehabilitation** |  |
| 1 | Abuzeyad 2020 | **Bahrain** | Evolution of emergency medical services in the Kingdom of Bahrain | X |  |  |  |
| 2 | Alrowayeh 2019 | **Kuwait** | Evidence-Based Physical Therapy Practice in the State of Kuwait: A Survey of Attitudes, Beliefs, Knowledge, Skills, and Barriers |  |  | X |  |
| 3 | Alhadhoud 2021 | **Kuwait** | The epidemiology of spinal fractures in a level 2 trauma center in Kuwait |  | X |  |  |
| 4 | Abuzeyad 2025 | **Kuwait** | The status of emergency medicine in the state of Kuwait | X | X |  |  |
| 5 | Al-Thani 2023 | **Qatar** | Trauma Quality Improvement Program: A Retrospective Analysis from A Middle Eastern National Trauma Center |  | X |  |  |
| 9 | Harthi 2024 | **Saudi Arabia** | The current status of trauma care for older adults in Saudi Arabia | X | X | X |  |
| 6 | Moafa 2022 | **Saudi Arabia** | Variation in on-scene time of emergency medical services and the extent of the difference of on-scene time between genders: a retrospective population-based registry study in Riyadh Province, Saudi Arabia | X |  |  |  |
| 8 | Alferdaus 2021 | **Saudi Arabia** | Current Trauma Care System in Saudi Arabia: Literature Review and a Proposed Action Plan | X | X | X |  |
| 11 | Mehmood 2017 | **Oman** | Development of an mHealth trauma registry in the Middle East using an implementation science framework |  | X |  |  |
| 7 | Alharbi 2015 | **Saudi Arabia** | A GIS-Based Decision Support System for Reducing Air Ambulance Response Times: A Case Study on Public Schools in Jeddah City | X |  |  |  |
| 10 | Al Busaidy 2012 | **Oman** | Occupational therapy in Oman: the impact of cultural dissonance |  |  | X |  |
| 13 | Alao 2023 | **UAE** | Trauma deaths of hospitalized patients in Abu Dhabi Emirate: a retrospective descriptive study |  | X |  |  |
| 12 | Koornneef 2017 | **UAE** | Progress and outcomes of health systems reform in the United Arab Emirates: a systematic review |  | X |  |  |
| 15 | Abuzeyad 2022 | **Mixed** | A Comparison of Three Emergency Medical Services Organizations in the Gulf Council Cooperation Countries | X |  |  |  |
| 14 | Abuzeyad 2021 | **Mixed** | The journey of emergency medicine in the Arabian Gulf States | X | X |  |  |
